# Mini Review: the non-neuronal cardiac cholinergic system in type-2 diabetes mellitus

**DOI:** 10.3389/fcvm.2024.1425534

**Published:** 2024-09-02

**Authors:** Eng Leng Saw, Martin Fronius, Rajesh Katare, Yoshihiko Kakinuma

**Affiliations:** ^1^ Whitaker Cardiovascular Institute, Boston University Chobanian & Avedisian School of Medicine, Boston, MA, United States; ^2^ Department of Physiology, HeartOtago, University of Otago, Dunedin, New Zealand; ^3^ Department of Bioregulatory Science, Graduate School of Medicine, Nippon Medical School, Tokyo, Japan

**Keywords:** non-neuronal cardiac cholinergic system, acetylcholine, cardiomyocytes, diabetes mellitus, glucose metabolism, angiogenesis

## Abstract

Diabetic heart disease remains the leading cause of death in individuals with type-2 diabetes mellitus (T2DM). Both insulin resistance and metabolic derangement, hallmark features of T2DM, develop early and progressively impair cardiovascular function. These factors result in altered cardiac metabolism and energetics, as well as coronary vascular dysfunction, among other consequences. Therefore, gaining a deeper understanding of the mechanisms underlying the pathophysiology of diabetic heart disease is crucial for developing novel therapies for T2DM-associated cardiovascular disease. Cardiomyocytes are equipped with the cholinergic machinery, known as the non-neuronal cardiac cholinergic system (NNCCS), for synthesizing and secreting acetylcholine (ACh) as well as possessing muscarinic ACh receptor for ACh binding and initiating signaling cascade. ACh from cardiomyocytes regulates glucose metabolism and energetics, endothelial function, and among others, in an auto/paracrine manner. Presently, there is only one preclinical animal model – diabetic db/db mice with cardiac-specific overexpression of choline transferase (*Chat*) gene - to study the effect of activated NNCCS in the diabetic heart. In this mini-review, we discuss the physiological role of NNCCS, the connection between NNCCS activation and cardiovascular function in T2DM and summarize the current knowledge of S-Nitroso-NPivaloyl-D-Penicillamine (SNPiP), a novel inducer of NNCCS, as a potential therapeutic strategy to modulate NNCCS activity for diabetic heart disease.

## 1 Introduction

Type-2 diabetes mellitus (T2DM), a chronic metabolic disorder characterized by hyperglycemia and insulin resistance, continues to grow in incidence and prevalence worldwide (1). Diabetic heart disease, referred to a collection of cardiovascular disease such as coronary artery disease, myocardial infarction, diabetic cardiomyopathy, and heart failure, is a major cause of mortality among individuals with T2DM [reviewed in (2)]. While the pathophysiology of diabetic heart disease is complex and yet to be clearly defined, insulin resistance and metabolic derangements contribute significantly [reviewed in (3)]. Both factors impair cardiac glucose metabolism (4–7) and coronary perfusion (8–11), as well as promotes myocardial fibrosis (12–14), contributing to cardiovascular dysfunction in diabetic individuals.

The parasympathetic nervous system exerts negative chronotropic, inotropic, and dromotropic effects on the heart, primarily mediated through acetylcholine (ACh) produced by cholinergic neurons [reviewed in (15)]. Subsequent studies have revealed that cardiomyocytes possess a cholinergic machinery capable of synthesizing and secreting ACh. This machinery, referred to as the non-neuronal cardiac cholinergic system (NNCCS), is crucial for auto/paracrine regulation of the heart [reviewed in (16)]. The presence of NNCCS is considered complementary to the parasympathetic nervous system, given the sparse cholinergic innervation in the ventricular myocardium (17–21). In the heart, other non-neuronal cardiac cells such as endothelial cells similarly possess this cholinergic machinery (22, 23), while smooth muscle cells, pericytes and fibroblasts have not been characterized or shown to possess cholinergic machinery (24). The primary source of non-neuronal ACh in the heart is likely cardiomyocytes, given their prevalence among cardiac cells (25).

NNCCS comprises key components such as the ACh-synthesizing enzyme (choline acetyltransferase, ChAT), vesicular ACh transporter (VAChT), choline transporter 1 (CHT1) and ACh-degrading enzyme (acetylcholinesterase, AChE). Furthermore, cardiomyocytes express multiple isoforms of muscarinic ACh receptor (MAChR), with the type-2 isoform (M_2_AChR) being predominantly expressed (26), enabling ACh binding and initiation of signaling transduction. The current understanding of the role of NNCCS in the heart includes the regulation of glucose metabolism, induction of angiogenesis, regulation of heart rate, anti-hypertrophy, and immunomodulation (27–33).

Dysregulation of NNCCS has previously been demonstrated in the left ventricle of patients with non-ischemic dilated cardiomyopathy (33) and diabetic heart disease (34). In this mini-review, we specially focus on the role of NNCCS in the diabetic heart in T2DM. We first discuss the physiological role of NNCCS and evidence of NNCCS dysregulation in the diabetic heart, followed by a discussion about the effect of activated NNCCS on the cardiac and vascular function demonstrated in diabetic db/db mice with cardiac-specific overexpression of *Chat* gene. Lastly, we discuss the therapeutic potential of S-Nitroso-NPivaloyl-D-Penicillamine (SNPiP), a novel nitric oxide donor and an inducer of NNCCS, to modulate NNCCS activity for treating diabetic heart.

## 2 Role of NNCCS in regulating normal physiology of the heart

The NNCCS maintains efficient energy metabolism in cardiomyocytes by promoting glucose-dependent metabolism and suppressing mitochondrial oxygen consumption and activity (28, 29, 35–37). Previous studies have shown that this energy regulatory role is mediated by the pro-survival phosphatidylinositol-3-kinase (PI3K)/protein kinase B (Akt)/hypoxia-inducible factor 1 (HIF)-α non-hypoxic signaling cascade induced by ACh, which results in an upregulation of glucose transporter (GLUT)-4 to enhance glucose uptake (28, 29, 38). GLUT-4 is noted as the predominantly expressed isoform of GLUT in the adult heart [reviewed in (39)]. Although cardiomyocytes primarily utilize free fatty acid for energy production, glucose is more energy-efficient, requiring few oxygen molecules for oxidation (phosphate/oxygen ratio = 2.58) compared to free fatty acid (phosphate/oxygen ratio = 2.33) [reviewed in (40)].

Furthermore, NNCCS regulates angiogenesis and maintains coronary vasculature in the heart. This role is similarly mediated by HIF-1α non-hypoxic signaling, leading to the upregulation of pro-angiogenic vascular endothelial growth factor (VEGF) in cardiomyocytes (27–29, 38). The secreted VEGF induces an angiogenic response in endothelial cells in a paracrine manner.

Additionally, NNCCS plays a crucial physiological role in modulating heart rate and contraction force by counteracting sympathetic stimulation (31, 33). While the underlying mechanism remains to be clearly elucidated, it is proposed that ACh released from cholinergic neurons (parasympathetic nervous system) stimulates ACh synthesis in cardiomyocytes through a positive feedback mechanism (35). The cardiac-derived ACh then acts on neighboring cardiomyocytes and other cardiac cells such as sinoatrial and atrioventricular node cells, facilitating the propagation of the neuronal cholinergic effect despite the sparse cholinergic innervation in the ventricular myocardium. Moreover, NNCCS is known to counteract sympathetic-induced cardiac hypertrophy and remodeling in response to stress (31–33, 41).

Multiple studies have demonstrated that NNCCS, similar to vagal nerve stimulation (42), helps maintain gap junctions by regulating the expression and localization of connexin 43 and β-catenin in cardiomyocytes (28, 37). Functional gap junctions are crucial for cell-cell communication and, most importantly, for propagation of action potential in the heart (43). This suggests that NNCCS is essential for maintaining the electrophysiological function of the heart. However, the precise mechanism by which NNCCS regulates connexin 43 and β-catenin has not yet been demonstrated.

**Figure 1:**
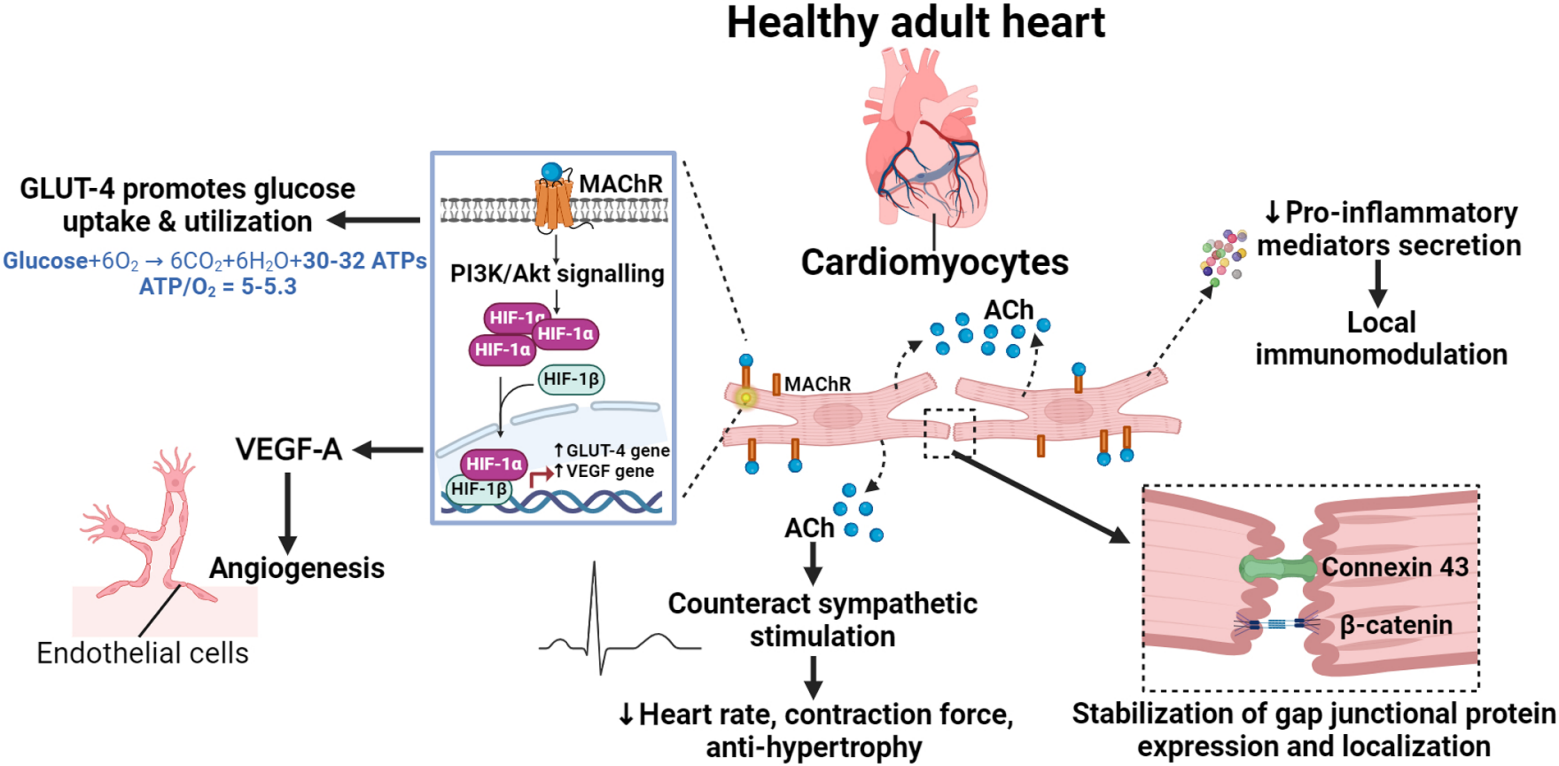
The physiological role of NNCCS in the healthy heart. Acetylcholine (ACh) released from cardiomyocytes initiates pro-survival PI3K/Akt/HIF-1α non-hypoxic signalling in an auto/paracrine manner, inducing the expression of glucose transporter (GLUT)-4 and the pro-angiogenic vascular endothelial growth factor (VEGF)-A. GLUT-4 promotes glucose uptake/utilization, leading to efficient energy metabolism, while VEGF-A induces angiogenic activity in endothelial cells. ACh from cardiomyocytes helps amplify neuronal cholinergic effects to modulate heart rate and contraction force, as well as to counteract sympathetic-induced cardiac hypertrophy and remodelling. ACh from cardiomyocytes maintains functional gap junctions by regulating the expression and localization of connexin 43 and β-catenin. Additionally, it also modulates the local inflammatory response by regulating the expression of pro-inflammatory mediators.

The immunomodulatory role of NNCCS is a newly discovered physiological function. NNCCS decreases the expression of pro-inflammatory mediators, including c-c motif chemokine ligand (CCL)-2 and -7, thereby preventing the influx of proinflammatory c-c motif chemokine receptor (CCR)-2-positive monocytes to the site of injury in the heart (32). This suggests that cardiomyocytes, at least in part, modulates local inflammatory responses through NNCCS and mediates paracrine interactions with inflammatory cells. However, the precise mechanisms by which NNCCS regulates expression of pro-inflammatory mediators remain unclear and warrant further investigation. A schematic illustration of the role of NNCCS in regulating normal physiology of heart is shown in Figure 1.

## 3 NNCCS in T2DM

A time-course study using diabetic db/db mice of different ages was conducted to assess the putative role of NNCCS during disease progression (34). In early stage of T2DM, characterized by hyperglycemia and insulin resistance in db/db mice (12–16 weeks old) (44), cardiac ACh levels (i.e., measured in the left ventricular lysate) remained unchanged despite decreased ChAT protein expression in the diabetic heart. In established (20–24 weeks old) and progressed stages of T2DM (28–32 weeks old) with deteriorating cardiovascular function (11, 45), cardiac ACh levels decreased along with reduced ChAT protein expression in the diabetic heart. This suggests that NNCCS dysregulation may play a role in the pathogenesis of cardiovascular dysfunction in diabetic hearts.

Interestingly, additional evidence suggested a compensatory effect in response to reduced cardiac ACh levels, as demonstrated by decreased AChE protein expression and increased CHT1 protein expression in the diabetic heart, specifically in progressed stage of T2DM (34). M_2_AChR protein expression was consistently reduced in the diabetic heart across early, established, and progressed stages of T2DM, indicating likely diminished NNCCS-mediated signaling.

Moreover, reduced cardiac ACh levels were accompanied by decreased cardiac glucose contents and GLUT-4 protein expression in the heart of db/db mice in established and progressed stages (34). This suggests that changes in cardiac ACh levels and/or NNCCS activity is associated with impaired glucose metabolism and may contribute to the development of cardiovascular dysfunction in T2DM. Besides these changes in db/db mice, reduced ChAT and CHT1 protein expressions were observed in the left ventricle biopsies from patients with T2DM (34), underscoring the putative clinical significance of NNCCS in T2DM-related cardiovascular disease.

While the mechanism behind NNCCS dysregulation in T2DM remains to be fully elucidated, *in vitro* studies showed that insulin upregulates ChAT expression in cultured human cardiomyocytes. Interestingly, this effect was abolished in cardiomyocytes exposed to palmitate to induce insulin-resistance [data published in supplemental results by Saw et al. (34)]. This study was the first to demonstrate insulin's effect on ChAT expression in cardiomyocytes, as was previously known only in neuronal cells (46–48). Further, these findings also suggest a potential link between hyperlipidemia and NNCCS dysregulation in T2DM, as hyperlipidemia can lead to excessive intracellular lipid accumulation and subsequently lipotoxicity, which is detrimental to cardiovascular function (49).

## 4 NNCCS and cardiac function in T2DM

**Figure 2:**
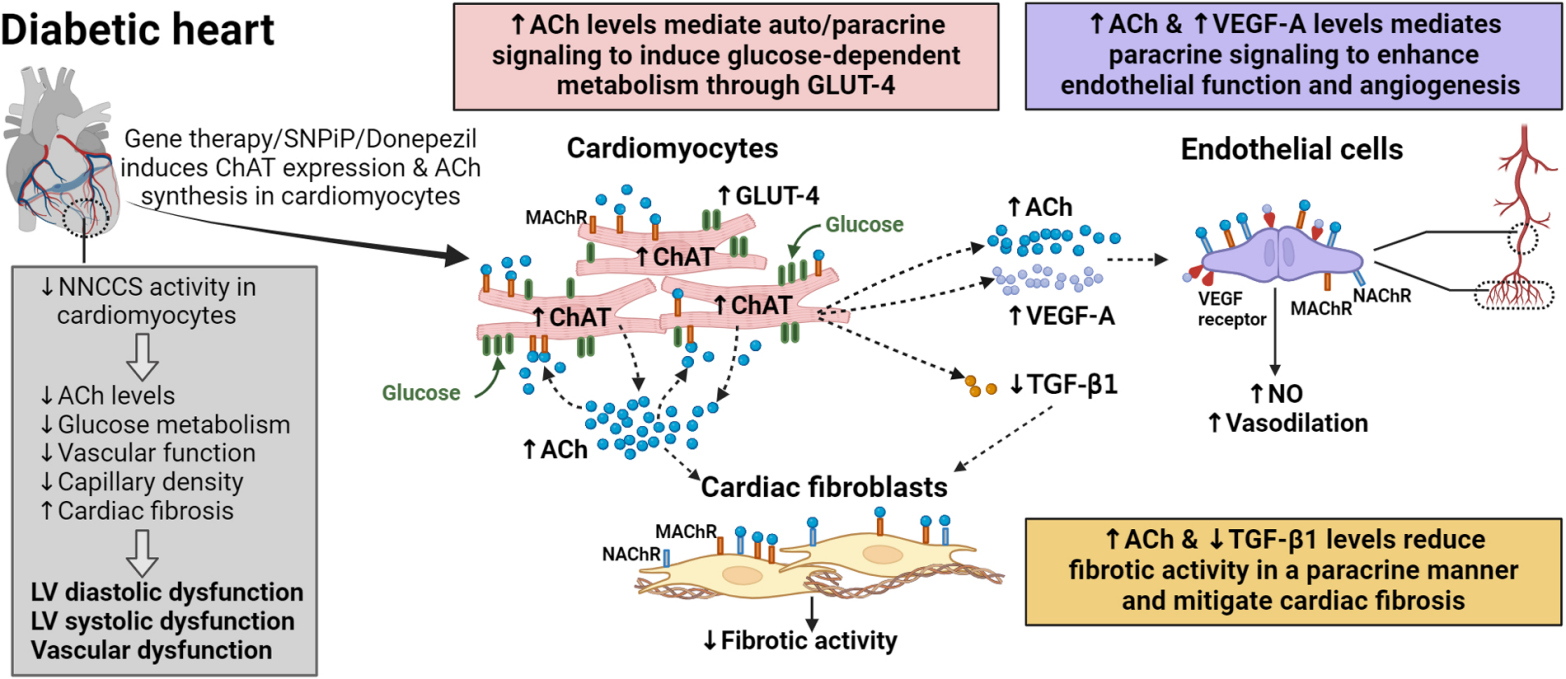
Auto/paracrine function of NNCCS in the diabetic heart. Reduced NNCCS activity is associated with diabetes mellitus-induced cardiovascular dysfunction. NNCCS activity in diabetic heart can be induced through gene therapy (cardiac-specific overexpression of the *Chat* gene) or pharmacological agents such as SNPiP and donepezil. Activated NNCCS leads to elevated acetylcholine (ACh) levels in diabetic cardiomyocytes, inducing the expression of glucose transporter (GLUT)-4, pro-angiogenic vascular endothelial growth factor (VEGF)-A, while diminishing the expression of pro-fibrotic transforming growth factor (TGF)-β1. In diabetic cardiomyocytes with activated NNCCS, GLUT-4 promotes glucose uptake/utilization, improving cardiac efficiency. Diabetic cardiomyocytes mediate paracrine signalling in endothelial cells through VEGF-A and ACh to enhance endothelial function and promote angiogenesis of coronary vessels and capillaries. Similarly, diabetic cardiomyocytes mediate paracrine signalling through ACh to attenuate the fibrotic activity of cardiac fibroblasts. Reduced TGF-β1 levels also diminish fibrotic activity in the diabetic heart.

In T2DM, cardiac dysfunction often remains asymptomatic and undetected in the early stage, marked by left ventricular (LV) hypertrophy and diastolic dysfunction [review in (3)]. As the disease progresses, cardiac dysfunction deteriorates further, leading to LV dilation and systolic dysfunction. This progression is accompanied by significant alterations in cardiac energy metabolism (4–7) and accelerated myocardial fibrosis (12–14) in diabetic hearts, contributing to cardiac dysfunction. Activation of NNCCS in diabetic hearts, as demonstrated in unique diabetic db/db mice with cardiac-specific overexpression of *Chat* gene (db/db-ChAT-tg mice), has been shown to improve cardiac function by mitigating alterations in cardiac energy metabolism and myocardial fibrosis (Figure 2) (34). This improvement is associated with augmented cardiac ACh levels as well as activation of ACh-mediated pro-survival PI3K/Akt/HIF-α non-hypoxic signaling cascade through MAChR in the heart of db/db-ChAT-tg mice (28, 29, 38).

Insulin resistance and metabolic derangement lead to a shift in substrate supply and utilization in cardiomyocytes, thereby altering cardiac energy metabolism in diabetic conditions. Increased fatty acid and decreased glucose metabolism were previously described, which may predispose the heart to the development of diastolic and systolic dysfunction (4–7). These metabolic changes occur early in T2DM (50), suggesting that early rectification of cardiac metabolism may prevent the development of diabetes-associated cardiac dysfunction (51, 52). In the heart of db/db-ChAT-tg mice and cultured human cardiomyocytes with overexpression of *CHAT* gene, activated NNCCS increased glucose contents, GLUT-4 protein expression and membrane translocation (34). This indicates a shift towards glucose utilization in diabetic cardiomyocytes, leading to greater cardiac efficiency (i.e., cardiac work/O_2_ consumed) and improved energy status (53). Upregulation of GLUT-4 protein expression is mediated by HIF1 transcription factor, as PI3K/Akt signaling prevents degradation of HIF-α, allowing it to dimerize with HIF-β to form HIF1 under normoxic conditions.

Accelerated myocardial fibrosis increases ventricular wall stiffness, contributing to diastolic dysfunction in the diabetic heart (12–14). Cardiac fibroblasts are the key extracellular matrix (ECM)-producing cells responsible for myocardial fibrosis (54). Their fibrotic activity can be activated by neighboring cells including cardiomyocytes, immune cells, endothelial cells, and others, through fibrogenic growth factors in the heart [reviewed in (55, 56)]. Activated NNCCS led to a reduction in pro-fibrotic transforming growth factor (TGF)-β1 protein expression and fibrotic area in heart of db/db-ChAT-tg mice (34). While various cell types can produce TGF-β1 (57), cardiomyocytes are likely the major source under pathological condition (58). However, ACh may also directly act on cardiac fibroblasts since these cells express MAChR and nicotinic ACh receptor (NAChR) (59), and a previous study showed that activation of M_3_AChR in cardiac fibroblasts led to anti-fibrotic effect through microRNA-29b/beta-site app cleaving enzyme 1 pathway (60).

## 5 NNCCS and vascular function in T2DM

In diabetic hearts, impaired endothelium-dependent vasodilation and capillary rarefaction disrupt coronary microcirculation, limiting the delivery of oxygen and nutrients to cardiomyocytes (8–11). Cardiac endothelial cells regulate vascular tone by producing the potent vasodilator nitric oxide (NO) in response to ACh binding on MAChR and NAChR (23, 61). Their proliferative activity can be mediated by pro-angiogenic factors including VEGF-A, which is essential for angiogenesis [reviewed in (62)].

Activation of NNCCS resulted in preserved endothelial function as well as an increase in the number of second order coronary vessels and capillary density, along with a concurrent increase in VEGF-A protein expression in the heart of db/db-ChAT-tg mice (Figure 2) (34). Elevated ACh levels from cardiomyocytes stimulate the synthesis of vasoactive factors in endothelial cells, including NO, that trigger vasodilation (23, 61), and also induce endothelial cell migration, a key event in angiogenesis, through NAChR (63). Upregulation of VEGF-A is induced by HIF1 transcription factor through ACh-mediated PI3K/Akt signalling cascade in cardiomyocytes. Notably, VEGF-A serves a dual role in angiogenesis induction and enhanced NO production by upregulating endothelial NO synthase (eNOS) expression in endothelial cells (64, 65). Moreover, since endothelial cells also possess cholinergic machinery like cardiomyocytes (22, 23), it is plausible that ACh from cardiomyocytes further stimulate endothelial cells to synthesize ACh in a positive feedback mechanism (35), thereby contributing to improved vascular function in the diabetic heart.

## 6 Targeting NNCCS as a possible therapy for diabetic heart disease

Despite emerging evidence demonstrating the beneficial effects of ACh against cardiovascular disease, there is currently no clinical therapy available to deliver or augment cardiac ACh levels in the heart. This makes NNCCS as a potential therapeutic target to achieve this goal. Possible approaches, such as vagal nerve stimulation, stem cell therapy, AChE inhibitors, gene therapy and microRNA therapy, have been summarized previously [reviewed in (16)].

Indirect evidence linking NNCCS with cardioprotection in clinical settings was previously demonstrated (66–69). These studies reported that patients with Alzheimer's disease treated with AChE inhibitors, including donepezil, experienced an improved survival rate, prognosis, and a reduced risk in cardiovascular events. Donepezil, an AChE inhibitor, was demonstrated to augment ACh synthesis in cultured primary rat cardiomyocytes (35), suggesting that donepezil can play a role in inducing NNCCS activation. Based on these basic and clinical results, donepezil may protect the heart from cardiovascular diseases, partly through NNCCS activation.

S-Nitroso-NPivaloyl-D-Penicillamine (SNPiP), a novel NNCCS inducer and also a NO donor (patent number is JP7338877 and available from FUJIFILM Wako Chemicals Corporation as product code 197-19151), has previously been shown to induce NNCCS activity in cellular models and mice (70). SNPiP activated NNCCS through NO/cyclic guanosine monophosphate (cGMP) signalling, leading to increased ChAT protein expression and cardiac ACh levels in the heart (70, 71). This resulted in enhanced cardiac output and diastolic function in healthy mice. A recent transcriptome analysis revealed that SNIPiP also activated various signalling pathways, namely hypoxic response pathway, cGMP pathway, and oxytocin signalling (71), and further, upregulated atrial natriuretic peptide and modulated calcium handling, known to have a cardioprotective effect [reviewed in (72)].

In diabetic db/db mice, SNPiP administration acutely improved diastolic and systolic function with no significant effect in heart rate after a single dose injection for five consecutive days (73). This indicates that SNPiP does not work like inotropic agents but directly improves cardiac relaxation and contraction through the Frank-Starling mechanism (71). However, thorough investigations are needed to evaluate the long-term safety and efficacy of SNPiP. While SNPiP has only been tested in db/db mice, it could potentially protect the heart against other cardiovascular diseases such as myocardial infarction, which is linked to decreased energy metabolism (28, 74), arrhythmia that is linked to electrical remodelling due to decreased connexin 43 (42, 75), thereby potentially slowing the progression of heart failure.

## 7 Conclusions

Activation of NNCCS offers significant advantages for the diabetic heart. It facilitates auto/paracrine communication among cardiomyocytes, cardiac fibroblasts, and endothelial cells within the diabetic heart, thereby improving cardiac metabolism, mitigating myocardial fibrosis, and preventing vascular rarefaction and endothelial dysfunction (Figure 1). Further research is warranted to delve deeper into these mechanisms and explore their potential therapeutic applications.
